# A Simple and Sensitive Method for Quantitative Measurement of Methylmalonic Acid by Turbulent Flow Chromatography and Tandem Mass Spectrometry

**DOI:** 10.4172/2157-7064.1000336

**Published:** 2016-09-21

**Authors:** AG Tecleab, RC Schofield, LV Ramanathan, Dean C Carlow

**Affiliations:** Department of Laboratory Medicine, Memorial Sloan Kettering Cancer Center, New York, NY 10065, USA

**Keywords:** Methylmalonic acid, Method validation, Mass spectrometry, Turbulent flow liquid chromatography, Protein precipitation

## Abstract

A simple and sensitive method for the detection of methylmalonic acid in serum without derivatization has been developed. This method implements protein precipitation using methanol followed by additional sample clean up by turbulent flow liquid chromatography (TFLC). The sample was directly injected into the turbulent flow liquid chromatography tandem mass spectrometry system (TFLC-MS/MS) for online extraction followed by HPLC separation. The eluent was transferred to the mass spectrometer and ionized by heated electrospray negative ionization (HESI) and the analyte was quantified using a six-point calibration curve. The validated analytical measurement range (AMR) is 30–1,000 nMol/L. Dilutions of 10 and 200-fold were validated giving a clinical reportable range (CRR) of 30–200,000 nMol/L. The between-day and within-day imprecision values at concentrations spanning the AMR were less than 15%. This method was compared to an established LC-MS/MS method at a CLIA certified national reference laboratory and shows an excellent correlation with our TFLC-MS/MS method.

## Introduction

Methylmalonic acid (MMA) levels in serum are commonly used as a marker for cobalamin (vitamin B12) deficiency or to diagnose patients with inherited defects in methylmalonyl CoA mutase enzyme activity. Lack of vitamin B12 causes an increase in the concentration of MMA, and its measurement in serum plays an important role in diagnosing B12 deficiency, which can lead to megaloblastic anemia and irreversible neurological disorders [1,2]. A mild elevation of MMA (>400 nMol/L) is an early indicator of vitamin B12 deficiency while a large elevation (>40,000 nMol/L) is indicative of methylmalonic acidemia, which is an inborn metabolic disorder [3].

Quantitative methods for the measurement of MMA have previously been described. These earlier methods used either protein precipitation [4], solid phase extraction [5–7], ultra-filtration [8–11], or chemical derivatization [12–14] which was followed by MS/MS or GC/MS. One of the major difficulties in measuring MMA is the presence of similar organic acids such as succinic acid (SA) which is present in much greater concentrations. SA is chromatographically difficult to separate from MMA and the mass spectra are very similar. These previously described methods require extensive sample preparation and lengthy extractions protocols and large sample volumes. The advantage of turbulent flow chromatography is that it allows for the injection of sample extract with minimal preparation. The turbulent flow column is packed with large porous particles which allow the retention of small molecules while large proteinaceous molecules are discarded into the waste thereby allowing the removal of large molecules, such as proteins, from the sample [15]. A previously described TFLC method for the quantitation of MMA has been published however it requires large specimen volume and a laborious multi wash SPE extraction prior to analysis [7]. In the present study we utilize turbulent flow chromatography to obviate the need for chemical derivatization and simplified the sample preparation procedure. The small sample was prepared by precipitating proteins using methanol followed by centrifugation and the supernatant was directly injected into the turbulent flow column. This method is rapid, simple to perform and provides an accurate and precise quantitative method for the measurement of MMA in serum.

## Materials and Methods

### Chemicals and reagents

Methylmalonic acid (MMA) was obtained from Sigma-Aldrich (Saint-Louis, MO, USA). Methylmalonic acid (1 mg/mL in acetonitrile) and deuterated methylmalonic acid (MMA-D_3_) at a concentration of 1 mg/ml in acetonitrile were obtained from Cerilliant (Round Rock, TX, USA). All compounds had a purity greater than or equal to 99%. Acetone, formic acid, methanol, acetonitrile, 2-propanol, and water (LCMS grade) were from Fisher Scientific (Fair Lawn, NJ, USA). Commercially available human serum that had been chemically treated to remove endogenous small molecules (DC Mass Spect Gold^®^, stripped human serum) was obtained from Golden West Biologicals, Inc, (Temecula, CA, USA). Ammonium acetate was from Fisher Scientific (Fair Lawn, NJ, USA). Succinic acid (SA) and deuterated SA were from Sigma-Aldrich (Saint-Louis, MO, USA).

### Instrument and analytical conditions

A Thermo Scientific Aria TLX-2 turbulent flow chromatography system (Franklin, MA, USA) comprised of a CTC analytics PAL auto sampler, a low-pressure mixing quaternary pump (loading pump), a high-pressure mixing binary pump (eluting pump) and a three-valve switching device unit (VIM) containing six-port valves were operated in accordance with manufacturer recommendations. An in-depth description of the system was previously published [16]. The triple quadrupole mass spectrometer was a Thermo Scientific TSQ Quantum Ultra (San Jose, CA, USA) and it implemented a heated electrospray ionization probe that was maintained at 380°C. The analysis was performed in a negative ionization mode with a spray voltage of 4500 V. Nitrogen was used as the sheath, auxiliary and ion sweep gas at 60, 15, and 2 arbitrary units, respectively. The system was operated in selected reaction monitoring (SRM) mode with argon as the collision gas at a pressure of 1.5 mTorr. The ion transfer tube was maintained at 235°C. The entire system was controlled using Aria 1.6.2 software. The Turbo Flow column used was a Cyclone Max^®^ (0.5 mm × 50 mm) from Thermo Scientific (San Jose, CA, USA) and the analytical column was an Allure Organic Acid^®^ 5μm (150 mm × 3.0 mm) from Restek (Bellefonte, PA, USA). The mobile phase consisted of loading pump A (water), loading pump B (water containing 1% formic acid), and loading pump C (acetonitrile, 2-propanol and acetone in 1:1:1 ratio). Eluting pump A contained 15.2 mM ammonium acetate in water containing 0.06% formic acid and eluting pump B contained methanol.

### Preparations of stock solutions, calibration standards and quality control samples

The internal standard (MMA-D_3_) was prepared by diluting the stock solution (100 μg/mL) to 50 ng/mL in methanol and stored at −20°C. Two stock solutions of MMA were prepared gravimetrically in methanol (1 mg/mL) and were used to prepare calibrators and quality control materials. MMA obtained from Sigma-Aldrich was used for calibrators while MMA from Cerilliant was used for quality controls, recovery and imprecision studies.

Calibrators and quality controls were prepared by spiking MMA into the stripped human serum. Prior to the addition of MMA the stripped human serum was tested to ensure that the concentration of endogenous MMA was below the lower limit of quantitation (LLOQ) of the assay. Briefly, the stripped human serum was spiked to obtain a concentration of 1000 nMol/L of MMA and then further diluted to obtain a six point calibration curve (500, 250, 125, 62.5, and 50 nMol/L). The three levels of controls were prepared by spiking MMA into the stripped human serum to yield concentrations of 800, 400, 100, and 30 nMol/L. Calibrators and controls were aliquotted and stored at −80°C.

### Sample preparation

Samples were prepared by protein precipitation using methanol. Calibrators, controls or patient specimen (100 μL) were aliquotted into 1.5 mL micro centrifuge tubes followed by the addition of 200 μL of methanol containing MMA-D_3_ (50 ng/mL). Subsequently, samples were vortexed mixed for five seconds and incubated on ice for five minutes. The samples were then centrifuged at 13,000 RPM for ten minutes. The supernatant (150 μL) was transferred into auto sampler vials containing inserts and 60 μL was injected into the system for analysis by TFLC-MS/MS.

### Method validation

The method was validated per U.S. Food and Drug Administration (FDA) [17], Clinical Laboratory Standards Institute (CLSI) [18] and Clinical Laboratory Improvement Amendments (CLIA) guidelines. The assay is fully validated for imprecision, accuracy, linearity, recovery, and carryover, specificity and matrix effects.

### Specificity

**Figure 1:**
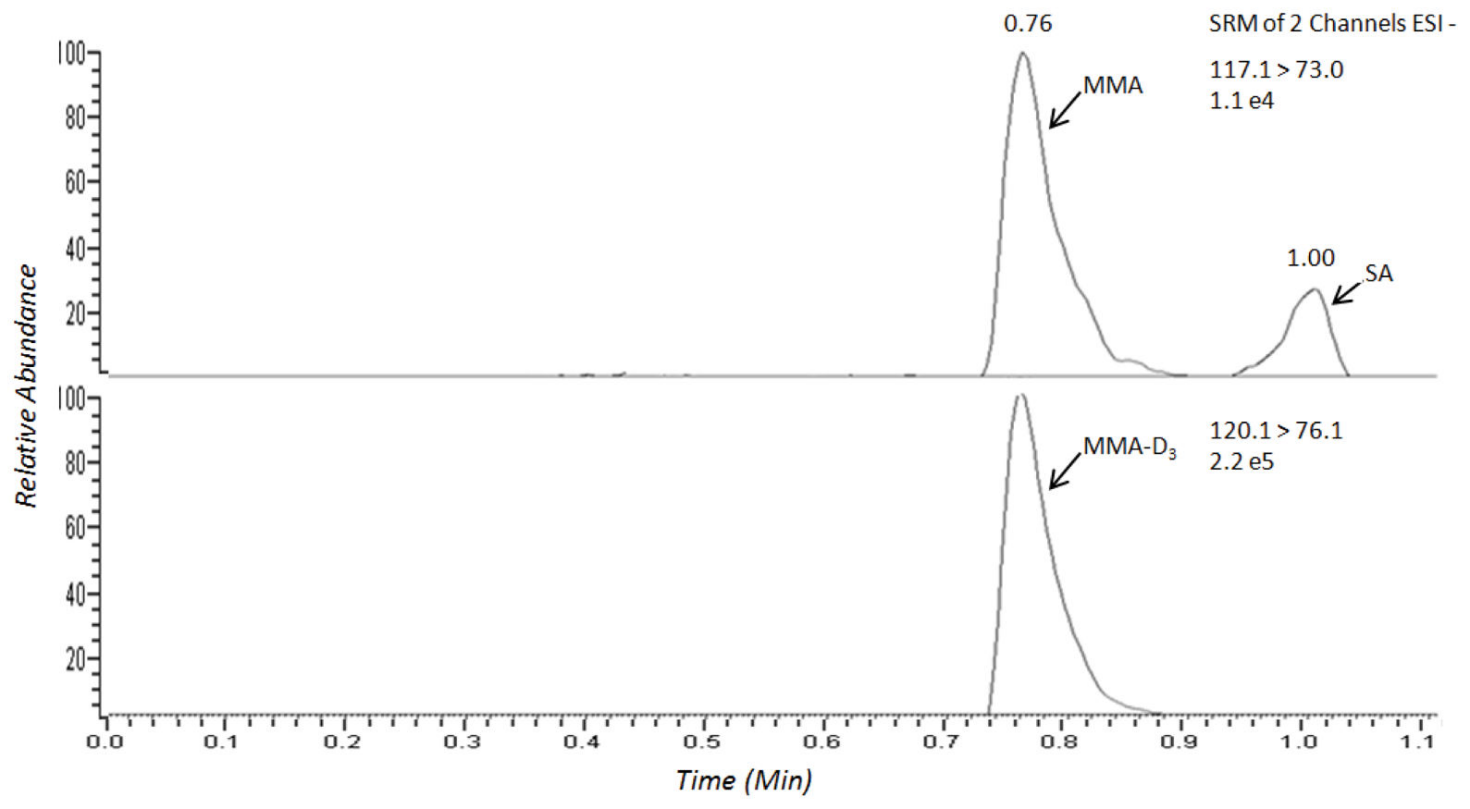
A normal patient TFLC-MS/MS ion chromatogram of MMA, MMA-D_3_, and SA.

The assay was validated for its ability to selectively detect MMA over SA, a compound which is found in patient samples at elevated concentrations and is difficult to separate chromatographically and is known to interfere with MMA in several assays. The specificity was tested by spiking both MMA and SA into stripped human serum and extracting the samples following the protocol described above. The chromatograms of both analytes were identified based on the SRM responses and retention times (Figure 1).

### Linearity

The linearity of this method was evaluated by using calibration curves as described in section 2.3. The calibration curves were generated by plotting the peak area ratios of MMA to the internal standard, MMA-D_3_. Weighted linear regression models with weights inversely proportional to the X values were used. The LLOQ was defined as the lowest concentration of analyte where the coefficient of variation (CV) was below 20%, per FDA guidelines [17]. In addition, the signal-to-noise ratio (S/N) of analyte at the LLOQs exceeded the minimum requirement [18].

### Accuracy and precision

Accuracy of the assay was assessed in two ways. The first way was by performing a recovery experiment, and additionally by comparing the results of 63 residual patient samples that were analyzed at a CLIA certified national reference laboratory using LC-MS/MS. Stripped human serum was spiked with MMA at three different concentrations spanning the analytical measurement range (AMR). Each level was then extracted in triplicate and the accuracy was calculated by determining the percent recovery. The correlation study was performed by extracting each patient sample as described above and comparing the results to the result obtained from the national reference laboratory and plotting the data using linear regression analysis.

A within-day imprecision study was conducted using stripped human serum spiked with MMA at concentrations of 30, 100, 400 and 800 nMol/L. For each level, ten separate extractions were prepared and analyzed in one day and the data was evaluated. Between-day imprecision was evaluated by extracting these same samples over 20 days in triplicate. The mean and standard deviation were determined over the validation period and the imprecision was calculated. The CV for the between-day imprecision should not be greater than 15% [17].

### Matrix interference and ion suppression

To evaluate matrix effects a post column infusion experiment was performed using an experimental set-up as described previously [16]. A tee was inserted between the outlet of the analytical column and a syringe was inserted into the tee to allow for the infusion of analyte into the mass spectrometer. A standard mixture solution of MMA and MMA-D_3_ were infused into the eluent stream at a flow rate of 10 μL/min. The signals of the corresponding MRM transitions of the analytes were recorded. After obtaining a steady baseline, a blank serum sample was extracted and injected and processed by the TFLC-MS/MS system. Any eluting compound that interfered with the ionization of target analytes would lead to an elevation or depression of the baseline which would represent matrix effects.

Several commonly occurring endogenous compounds which have the potential to interfere with assays were evaluated. Interferences from lipemic, hemolyzed, and icteric samples were tested by spiking MMA into patient samples containing high levels of triglyceride (926 mg/dL), hemoglobin (4.6 g/dL), and bilirubin (34.9 mg/dL). MMA was measured before and after the addition of MMA and the effect of the indices was assessed by subtracting the basal value from concentration determined after the addition of MMA.

### Quantitation of sample carryover

Sample carryover can be an issue with on-line sample preparation systems. To rule out carryover a blank stripped serum sample was injected after the highest calibrator during the validation period to confirm it did not exceed 20% of the LLOQ [18].

## Results and Discussion

### Chromatographic conditions

The turbulent flow chromatography parameters were adapted from Yuan et al. [7] and optimized to maximize sensitivity for all analytes. It was determined that the combination of a Cyclone-MAX (50 mm × 0.5 mm) and an Allure Organic Acids column (3 mm × 150 mm) produced adequate retention and separation of the compounds. Various mobile phase compositions, flow rates and profiles were evaluated. The desired sensitivity was achieved using water and water containing 0.1% formic acid in the quaternary pump for the loading and eluting solvents, respectively. While 15.2 mM ammonium acetate containing 0.06% formic acid in water and methanol were used in the binary pump for the loading and eluting solvents, respectively. The analytes were loaded on the turbulent flow column in 100% mobile phase A and transferred to the HPLC column with 100% mobile phase B using a 200 μL transfer loop. The loading and eluting mobile phase composition for the HPLC column are depicted in Table 1. The optimal injection volume was 60 μL and the analytical column was maintained at 70°C. The integration parameters for all analytes were similar with a baseline window of 50, area noise factor of 5, peak noise factor of 10 and an integration window of 15 seconds (Table 1).

### MS/MS detection

The TFLC-MS/MS analysis was performed as described in section 2.2. The optimization of the SRM parameters was executed by direct infusion of the standards using negative electrospray ionization. The transitions monitored for MMA and MMA-D_3_ in SRM mode were 117>73 and 120>76, respectively. Collision induced dissociation (CID) mass spectra were recorded. The optimal collision energies (CE) and tube lens values were 10 and 85 V, respectively. The skimmer offset was determined in MS mode to be optimal at a value of 10 V. The scan time was 0.05 (s) and scan width was 0.05 (m/z). The data was processed using LCquan software version 2.6.

### Method validation

As previously stated the method was validated per U.S. Food and Drug Administration [17], Clinical Laboratory Standards Institute [18] and CLIA guidelines. The assay is fully validated for imprecision, accuracy, linearity, recovery, and carryover, specificity and matrix effects.

### Specificity

A bioanalytical method should be selective for a specific analyte and not be affected by interfering or co-eluting components in a biological matrix. A TFLC-MS/MS SRM chromatogram for MMA, MMA-D_3_, and SA in human serum is shown in Figure 1. The retention times for MMA, MMA-D_3_, and SA were 0.76, 0.76, and 1.0 minutes, respectively. It can be observed in the chromatogram that separation of MMA from the isobaric interference from SA was achieved (Figure 1).

### Linearity

A linear relationship was found between analyte concentrations and peak area ratios throughout the AMR. The validated AMR of this method is 30–1,000 nMol/L. The coefficient of correlations (r) as determined by a six-point calibration curve was greater than >0.995. The LLOQ for this assay was determined to be 30 nMol/L. The signal-to-noise (S/N) of MMA at the LLOQ was 118; more than 5 times the S/N requirement with a between-day CV of <20% [17].

### Accuracy and precision

To assess the accuracy and precision of this method, QC samples at three different concentrations spanning the AMR were analyzed; 100, 400, and 800 nMol/L. The concentration of MMA in each sample was determined by comparing MMA concentration to internal standard response based on the calibration curve. The imprecision was calculated as the %CV for both the within-day and between-day batches. The within-day and between-day imprecision for the assay was less than 15% for the three levels of controls while the %CV at the LLOQ was less than 20% (Table 2).

The accuracy of the assay, as evaluated by the recovery experiments, resulted in a recovery of MMA ranging between 95.8 to 110.9% (Table 3).

We developed and validated a dilution protocol to allow the measurement of patient specimens with values greater than 1000 nMol/L. Specimen containing MMA at 100,000 and 5,000 nMol/L were diluted 200 and 10-fold, respectively. The data showed <7% deviation from the expected values.

### Correlation to alternative methods

**Figure 2:**
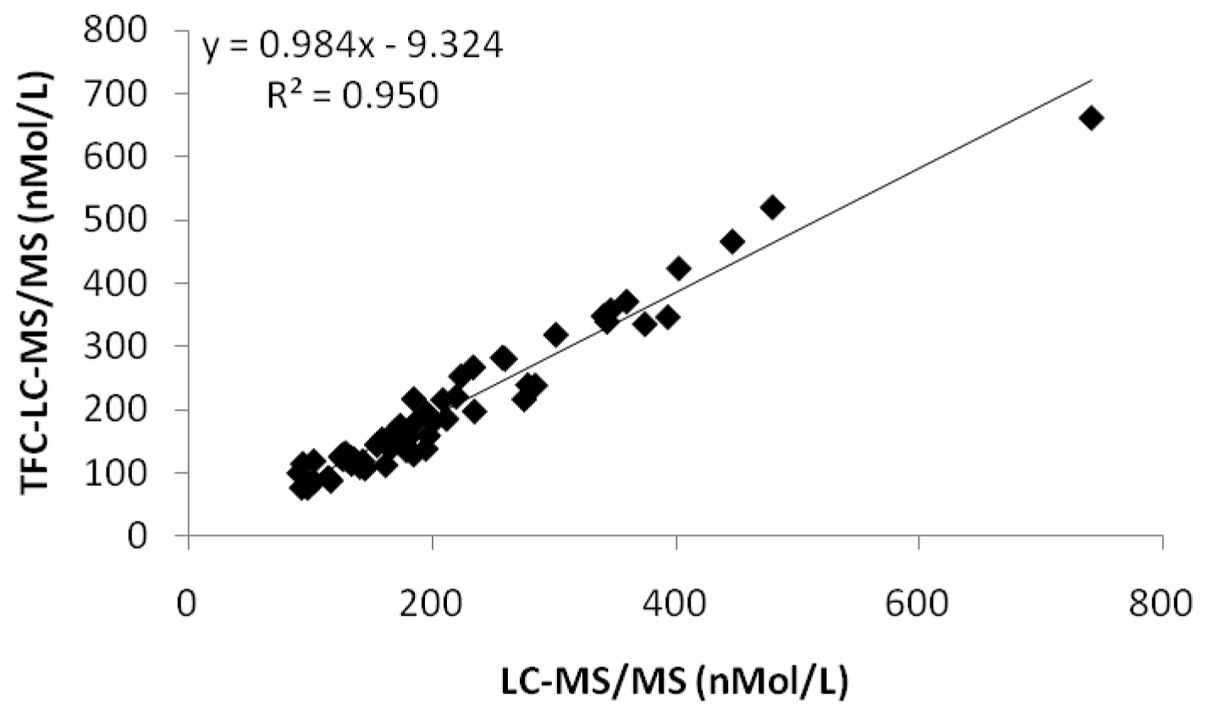
The TFLC-MS/MS assay for MMA compared with an LC-MS/MS assay from a CLIA certified national reference laboratory.

We further analyzed the accuracy of the assay by comparing the results from 63 patients to a LC-MS/MS method from a CLIA certified national reference laboratory. The slope of the linear regression curve was within 2% and exhibited an excellent correlation coefficient as shown in Figure 2.

### Matrix interference and ion suppression

Evaluation of possible matrix interference and ion suppression was conducted using a tee-infusion experiment which did not detect ion suppression or enhancement at the point of elution. We have also ruled out interferences from common endogenous compounds that can potentially cause interferences. No interferences were observed from triglycerides (926 mg/dL), bilirubin (34.9 mg/dL), or hemoglobin (4.6 g/dL).

### Quantitation of sample carryover

Sample carryover was evaluated by running a serum blank after the highest calibrator on all calibration curves during the validation period. The average carryover was determined to be less than 20% of the calculated response of the LLOQ. As mentioned previously the LLOQ of this assay is 30 nMol/L with a S/N greater than five times the minimal requirement.

## Conclusion

This article describes the development and validation of a simplified TFLC-MS/MS method for the quantification of MMA in human serum. TFLC for analyte extraction allows for reduced sample preparation and sample clean-up. Other methods have used solid phase extraction, ultra-filtration or derivatizations which are subject to elaborate procedures and additional instrumentation. This method allows for a simple protein precipitation which yields the required sensitivity for clinical applications. The assay is fully validated for specificity, sensitivity, accuracy, precision, linearity, and recovery. The method is accurate, with recoveries ranging between 96 and 111% at concentrations spanning the AMR and shows excellent agreement with an alternate LC-MS/MS assay form a CLIA certified national reference laboratory. The MMA assay is linear between 30–1000 nMol/L, and has excellent performance characteristics. There are no matrix interferences observed or interferences from other co-eluting compounds such as SA. Unlike other LC-MS/MS assays that require extensive sample preparation techniques we have development an application Utilizing turbulent flow chromatography that implements a simple protein precipitation to achieve the clinically required performance characteristics.

## Figures and Tables

**Table 1:** Gradient used on the loading and eluting pumps for the TFLC-LS/MS analysis of MMA.

Step	Time (s)	Flow (mL/min)	%A	%B	%C	Flow (mL/Min)	Grad.	%A	%B
1	30	3.00	100	-	-	0.8	Step	92	8
2^1^	30	0.90	100	-	-	0.2	Step	92	8
3^2^	90	2.00	-	50	50	0.8	Step	92	8
4	20	2.00	-	50	50	0.8	Step	50	50
5	40	3.50	-	100	-	0.8	Step	50	50
6	20	3.50	-	100	-	0.8	Step	92	8
7	120	2.00	100	-	-	0.8	Ramp	92	8
8	10	3.00	100	-	-	0.8	Ramp	92	8

1 During this step valve “A” and valve “B” are in line and the analyte is transferred from the turbulent flow column to the analytical column.

2 Data Acquisition window: 1.5–3.0 minutes.

**Table 2:** Within and between-day precision of MMA in human serum.

Sample	Nominal value (nMol/L)	Mean (nMol/L) Within-Day	Within-day CV (%) n=10	Mean (nMol/L) Between-Day	Between-day CV (%) n=20
LLOQ	30	29.8	19.4	32.8	19.4
Level 1	100	89.3	11.6	107.8	8.8
Level 2	400	415.2	2.8	414.5	5.0
Level 3	800	825.1	4.6	796.3	5.8

**Table 3:** Method accuracy.

Sample	Nominal value (nMol/L)	Measured value (nMol/L)	%Recovery (measured/nominal)
Low	100.0	95.8 ± 13.3	95.8 ± 13.9
Medium	500.0	531.7 ± 11.6	106.3 ± 2.2
High	800.0	887.2 ± 78.4	110.9 ± 8.8

## Abbreviations

AMR: Analytical measurement range
CLIA: Clinical laboratory improvement amendments
CRR: Clinical reportable range
HESI: Heated electrospray ionization
LLOQ: Lower limit of quantitation
MMA: Methyl malonic acid
MMA-D3: Deuterated methyl malonic acid
SA: Succinic acid
SRM: Selected reaction monitoring
TFLC: Turbulent flow liquid chromatography
